# Comparison of extracorporeal and conventional cardiopulmonary resuscitation: a retrospective propensity score matched study

**DOI:** 10.1186/s13054-019-2320-1

**Published:** 2019-01-28

**Authors:** Daniel Patricio, Lorenzo Peluso, Alexandre Brasseur, Olivier Lheureux, Mirko Belliato, Jean-Louis Vincent, Jacques Creteur, Fabio Silvio Taccone

**Affiliations:** 1 Department of Intensive Care, Erasme Hospital, Université Libre de Bruxelles (ULB), Route de Lennik, 808, 1070 Brussels, Belgium; 2 U.O.C. Anestesia e Rianimazione 1, Fondazione IRCCS Policlinico San Matteo, Pavia, Italy

**Keywords:** Extracorporeal cardiopulmonary resuscitation, ECMO, Out-of-hospital, Survival rate, Neurological outcome, Post-anoxic brain damage

## Abstract

**Background:**

The potential benefit of extracorporeal cardiopulmonary resuscitation (ECPR) compared to conventional CPR (CCPR) for patients with refractory cardiac arrest (CA) remains unclear.

**Methods:**

This study is a retrospective analysis of a prospective database of CA patients, which includes all consecutive adult patients admitted to the Department of Intensive Care after CA between January 2012 and December 2017. The decision to initiate ECPR was made by the attending physician and ECPR performed by the ECPR team, which is composed of ICU physicians. A propensity score was derived using a logistic regression model, including characteristics that varied between groups with a *p* <  0.10 and were potentially related to outcome. Primary outcomes were survival to ICU discharge and favorable 3-month neurologic outcome, assessed by a Cerebral Performance Category (CPC) score of 1–2.

**Results:**

From a total of 635 patients with CA during the study period (ECPR, *n* = 112), 80 ECPR patients were matched to 80 CCPR patients. The time from arrest to termination of CPR (i.e., return of spontaneous circulation [ROSC], extracorporeal membrane oxygenation [ECMO] initiation, or death) was 54 ± 22 and 54 ± 19 min in the ECPR and CCPR groups, respectively. ROSC rates were 77/80 (96%) for ECPR and 30/80 (38%) for CCPR (*p* <  0.001). Survival to ICU discharge was 18/80 (23%) vs. 14/80 (18%) in the ECPR and CCPR groups, respectively (*p* = 0.42). At 3 months, 17/80 (21%) ECPR patients and 9/80 (11%) CCPR patients had a favorable outcome (*p* = 0.11). Cox regression analysis stratified by matched pairs showed a significantly higher neurologic outcome rate in the ECPR group than in the CCPR group (log-rank test *p* = 0.003).

**Conclusions:**

ECPR after CA may be associated with improved long-term neurological outcome.

## Introduction

Since the first use of cardiopulmonary resuscitation (CPR), remarkable technological and scientific progress has been made in this field. Interventions, such as early defibrillation and implementation of targeted temperature management (TTM), have helped improve outcomes for patients with CA [1, 2]. However, the rates of survival to hospital discharge of these patients remain low. Recent studies have shown a survival rate of around 20% for patients after in-hospital CA (IHCA) and 10% after out-of-hospital CA (OHCA) [3]. These findings may vary depending on the specific emergency medical system (EMS) in use and are essentially based on use of conventional CPR (CCPR).

“No-flow” (i.e., time from arrest to the first chest compression) and “low-flow” (i.e., duration of CCPR) times are important determinants of patient outcome following CA. Immediately initiated bystander CPR remains imperative for reducing no-flow time. Guidelines have highlighted the importance of telephone-assisted bystander guidance in providing prompt chest compressions to CA victims [1]. The early arrival of EMS staff to provide CPR and advanced life support (ALS) can reduce the low-flow time. Nevertheless, when CCPR is prolonged, even if adequately delivered, the probability of return of spontaneous circulation (ROSC) progressively decreases. In one study, Mosca et al. reported that CPR maneuvers lasting for more than 45 min were associated with hospital survival of less than 2%, most of the patients dying without achieving ROSC [4]. In this setting, in particular for patients who have received the best possible resuscitation (i.e., short no-flow time, bystander CPR, shockable rhythms refractory to defibrillation), there would be a high possibility of good cardiac and neurological recovery in case of ROSC, which make these patients as the best potential candidates for extracorporeal cardiopulmonary resuscitation (ECPR).

In patients with CA, ECMO may provide greater global blood flow than CCPR and thus possibly decrease post-anoxic cellular damage [3, 5]. ECPR could also be considered as a “bridge” to appropriate treatment (i.e., coronary angiography or cardiac surgery) of the underlying cause of CA, which would not be feasible during CCPR [6]. With ECPR, the patient’s heart is put “at rest,” allowing for partial or full recovery and minimizing the need for vasopressors and/or inotropic agents. Reversible causes of CA can be more easily identified and treated, if applicable. This approach may also translate into improved neurological outcomes because the total anoxic time is reduced and cerebral circulation guaranteed by the ECMO blood flow. In some selected cases of refractory CA, implementation of ECPR has increased survival rates up to 45% for IHCA and 30% for OHCA [3]. Moreover, survival rates of 33% for IHCA and 37% for OHCA have been reported with ECPR in patients with CA of cardiac origin and as a result of drug intoxication [7]. Nevertheless, although ECPR is easily administered for patients with IHCA, when the equipment if available, its use for patients with OHCA remains problematic and its efficacy in this setting is still controversial.

The aim of this study was to compare the effects of ECPR on survival and long-term neurological outcome when compared to CCPR using a propensity score matching method.

## Methods

### Study population

In this retrospective study, patients were selected from an institutional database of CA patients treated at Erasme University Hospital, a tertiary care center in Brussels, Belgium, whose EMS system covers around 250,000 inhabitants. The study protocol was approved by the Ethical Committee of the hospital (P2018/204), and informed consent was waived because of the retrospective nature of the analysis.

### Study setting

Since January 2012, a patient’s eligibility for ECPR after an incoming call from the EMS ambulance or after an IHCA is assessed by the physician on duty leading the ECMO team based on the following factors: age < 65 year, witnessed arrest, < 2 min of estimated no-flow time, < 75 min of estimated time to ECMO placement, no severe comorbidity, and signs of life during CPR [8]. Mechanical CPR (LUCAS Chest Compression System, Physio-Control Inc., Redmond, WA, USA) was routinely used in all CA patients with ongoing resuscitation which would fail the first 3 cycles of CPR. If indicated, ECMO is implanted by the ECPR team, which includes well-trained ICU physicians and/or cardiac surgeons. A peripheral femoro-femoral V-A percutaneous cannulation guided by echocardiography is used under mechanical CPR. Post-resuscitation care includes adequate oxygenation (i.e., PaO_2_ 80–150 mmHg), vasopressors and fluids to maintain mean arterial pressure (MAP) > 65–70 mmHg, therapeutic hypothermia (aiming at a body temperature of 34 °C for 24 h), and early cardiac catheterization when indicated. Anticoagulation is initiated using unfractionated heparin at 24 h after arrest to minimize the risk of bleeding. ECMO configuration includes a 25 Fr venous cannula and 18–22 Fr arterial cannula (Medtronic, Minneapolis, MN, USA). A centrifugal blood pump (Revolution Blood Pump, Sorin, Milan, Italy) is initially set at a blood flow of 3–4 L/min. ECMO priming consists of 700 mL of PlasmaLyte solution (Baxter Healthcare Corporation, Deerfield, USA). At the end of the implantation, the leg is perfused with an anterograde single lumen 8 Fr catheter (Arrow Inc., Reading, PA, USA) to prevent limb ischemia.

### Data collection

All patients with IHCA or OHCA of any cause were included in an institutional database between January 2012 and December 2017. Only patients admitted to the ICU were considered, because for patients dying on site during CPR for OHCA or on the ward without ICU admission, pre-ROSC data were not available. Patients with “Do-Not-Resuscitate” orders established prior to the CA and patients pronounced dead before hospital arrival were also excluded. Use of ECPR was crosschecked using an institutional ECMO database.

Use of ECPR or CCPR was noted. Demographics and comorbid diseases (i.e., hypertension, diabetes, respiratory disease, history of ischemic cardiac disease, pre-existing cardiac or renal failure, liver cirrhosis, or previous neurological diseases, which could have caused cognitive or other neurovascular disturbance) were recorded. Resuscitation factors were also noted: location and cause of cardiac arrest, primary cardiac rhythm reported by the rescue team, witnessed arrest, bystander CPR, and epinephrine doses. Time to ROSC was defined as the time to ROSC in CCPR, time to ECMO implementation in ECPR, and time to death when resuscitation attempts were stopped. For patients achieving ROSC, lactate levels on ICU admission, use of vasopressors or renal replacement therapy (RRT), and massive bleeding (defined as the use of at least four red blood cell units over 24 h because of a reduction by 2 g/dL in baseline hemoglobin) were recorded. Causes of death were separated into “neurological” (i.e., brain death or severe post-anoxic brain damage) or “cardiac/multiple organ failure” (i.e., severe cardiogenic shock, no cardiac recovery after ECPR, or multiple organ failure). The two main study outcomes were survival to ICU discharge and favorable neurological outcome at 3 months after CA, evaluated using the Cerebral Performance Category (CPC) scale: specifically, a score of 1 or 2 was considered a favorable outcome, and a score of 3 to 5 indicated an unfavorable neurological outcome or death. The time to death was also recorded. Survival and favorable outcome were also analyzed according to different times to ROSC (i.e., < 45 min, 45–60 min, and > 60 min) in both groups, to location of arrest (OHCA vs. IHCA), to initial rhythm (shockable vs. non-shockable), and to cause of arrest (cardiac vs. non-cardiac). Two secondary outcomes were considered: (a) the rate of sustained ROSC, defined as a pulse not requiring additional CPR for at least 60 min; (b) the number of patients who were suitable for organ donation, either because of brain death or because of irreversible brain damage and circulatory death (i.e., category III of the Maastricht definition) [9].

### Statistical analyses

To compare ECPR and CCPR, a pairwise matching procedure was used to reduce the effects of selection bias and possible confounding factors between groups. A propensity score was derived from a non-parsimonious logistic regression model that included all baseline pre-hospital characteristics that varied between the ECPR and CCPR groups by a *p* value less than 0.10 (i.e., age, gender, witnessed arrest, time to ROSC, non-cardiac origin of arrest, hypertension, diabetes, COPD/asthma, and previous neurological diseases), as previously published [10]. Also, other variables potentially related to outcome (i.e., OHCA vs IHCA, bystander CPR, and initial rhythm) were included into this model. Each patient was assigned a propensity score reflecting the probability of receiving ECPR. ECPR and CCPR cases were matched by their propensity score in blocks of 1:1, 1:2, and 1:3; the selected patients formed well-matched 1:1 pairs. The quality of matching was assessed by calculation of the standardized mean difference (SMD) between selected variables, with a SMD <  0.10 reflecting good matching. After the analysis of the matched population, non-cardiac origin of arrest was not retained in the final model to avoid a significant reduction of the examined cohort.

Continuous variables are expressed as mean ± standard deviation and were compared using a Student’s *T* test. Categorical variables, such as pre-admission demographics, were compared using chi-square or Fisher’s exact test. Survival with favorable outcome was defined as time from CPR to recovery at 3 months after CA, with survivors without favorable outcome being censored at ICU discharge. Differences in outcomes (survival and neurologic outcome at 3 months) between ECPR and CCPR were tested using conditional logistic regression for binary data. A Kaplan-Meier analysis plot was used to compare the proportions of survivors or those with favorable outcome in the two groups over the 3 months following CA. However, differences in the time to the occurrence of each outcome (i.e., survival and neurological outcome at 3 months) between ECPR and CCPR were tested using stratified log-rank test and were quantified using Cox regression analysis stratified by matched pairs. All the statistical analyses for this study were processed on IBM SPSS Statistics Version 24 (IBM Corporation). *p* values are two-tailed and values < 0.05 were considered statistically significant.

## Results

### Full study population

**Fig. 1:**
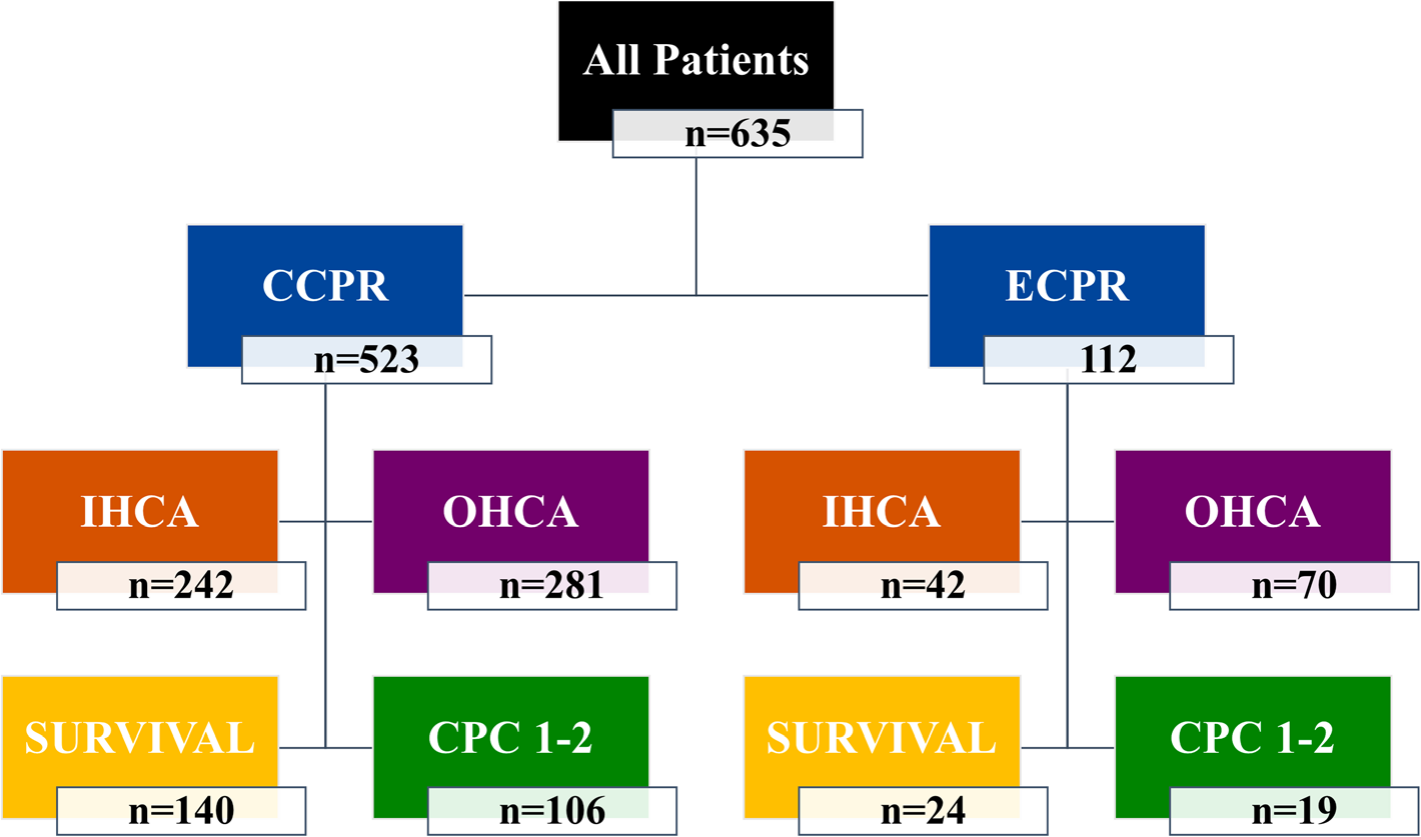
Flow chart of the study population

A total of 635 patients—112 in the ECPR group and 523 in the CCPR group—were admitted to the hospital after CA between January 2012 and December 2017 (Fig. 1). The baseline characteristics of the two groups are shown in Table 1. All patients in the ECPR group underwent ECMO cannulation on admission. Patients undergoing ECPR were younger and less likely to have previous neurological disease, hypertension, chronic obstructive pulmonary disease (COPD), asthma, or diabetes. They had a higher rate of witnessed CA and were more likely to have a cardiac origin of arrest. These patients also had a higher ROSC rate than CCPR patients. The time to ROSC was longer in the ECPR than in the CCPR group. Rates of survival and 3-month favorable outcome were similar between groups (21% vs. 27% and 16% vs. 20%, respectively).

**Table 1:** Demographic data of the study population

	All patients (*n* = 635)	CCPR (*n* = 523)	ECPR (*n* = 112)	*p* value
Age, years	62 ± 16	65 ± 16	54 ± 16	< 0.001
Male, *n* (%)	419 (66)	336 (64)	83 (74)	0.04
Witnessed CA, *n* (%)	480 (78)	387 (77)	93 (83)	0.04
Bystander CPR, *n* (%)	423 (68)	346 (68)	77 (69)	0.60
ROSC, *n* (%)	512 (81)	404 (77)	108 (96)	< 0.001
Time to ROSC, min	24 ± 21	18 ± 14	48 ± 27	< 0.001
Out-of-hospital CA	351 (55)	281 (54)	70 (63)	0.09
Non-cardiac origin CA	325 (51)	283 (54)	42 (38)	0.001
VF/VT, *n* (%)	176 (28)	141 (27)	35 (32)	0.36
Chronic heart failure, *n* (%)	139 (22)	114 (22)	25 (22)	0.89
Hypertension, *n* (%)	285 (45)	245 (47)	40 (36)	0.03
Coronary artery disease, *n* (%)	220 (35)	179 (34)	41 (37)	0.63
Diabetes, *n* (%)	137 (22)	123 (24)	14 (13)	0.01
COPD/asthma, *n* (%)	131 (21)	119 (23)	12 (11)	0.004
Neurological disease, *n* (%)	102 (16)	95 (18)	7 (6)	0.002
Chronic renal disease, *n* (%)	102 (16)	88 (17)	14 (13)	0.26
Liver cirrhosis, *n* (%)	43 (7)	37 (7)	6 (5)	0.52
TTM, *n* (%)	339 (54)	251 (48)	88 (79)	< 0.001
MV, *n* (%)	635 (100)	523 (100)	112 (100)	1.00
ICU stay, days	5 [1–6]	4 [1–90]	8 [1–90]	< 0.001
ICU survival, *n* (%)	164 (26)	140 (27)	24 (21)	0.28
3-month favorable neurological outcome, *n* (%)	111 (18)	106 (20)	19 (16)	0.48

*ICU* intensive care unit, *CA* cardiac arrest, *CPR* cardiopulmonary resuscitation, *ROSC* return of spontaneous circulation, *VF/VT* ventricular fibrillation/ventricular tachycardia, *COPD* chronic obstructive pulmonary disease, *ECPR* extracorporeal cardiopulmonary resuscitation, *MV* mechanical ventilation, *TTM* targeted temperature management

### Propensity score matched groups

Propensity score analysis matched 80 patients from the ECPR group to 80 patients from the CCPR group. SMDs for each variable are shown in Table 2; all variables were well matched, except for gender (ECPR 74% male vs. CCPR 61% male; SMD = 0.12) and cardiac origin of arrest (ECPR 72% vs. CCPR 45%; *p* <  0.001). Patients in the matched ECPR group more frequently had coronary artery disease; they also had a longer ICU stay. Blood lactate levels on admission were significantly higher in the ECPR than in the CCPR group.

**Table 2:** Demographic data of the matched (1:1) population

	CCPR (*n* = 80)	ECPR (*n* = 80)	*SMD*	*p* value
Age, years	57 ± 17	57 ± 14	0.01	0.93
Male, *n* (%)	49 (61)	59 (74)	0.08	0.13
Witnessed CA, *n* (%)	68 (85)	70 (88)	0.01	0.82
Bystander CPR, *n* (%)	59 (74)	57 (71)	0.05	0.86
ROSC, *n* (%)	30 (37)	77 (96)		< 0.001
Time to ROSC, min	54 ± 22	54 ± 20	0.004	
Out-of-hospital CA	50 (63)	49 (61)	0.03	1.00
Non-cardiac origin CA	44 (55)	22 (28)		< 0.001
Initial rhythm				
VF/VT, *n* (%)	23 (28)	24 (30)	0.09	0.93
Asystole, *n* (%)	38 (48)	35 (44)	0.10	0.75
PEA, *n* (%)	19 (24)	21 (26)	0.09	0.85
Chronic heart failure, *n* (%)	9 (11)	19 (24)		0.06
Hypertension, *n* (%)	30 (38)	30 (38)	0.002	1.00
Coronary artery disease, *n* (%)	20 (25)	33 (41)		0.05
Diabetes, *n* (%)	13 (16)	10 (13)	0.09	0.65
COPD/asthma, *n* (%)	11 (14)	8 (10)	0.08	0.63
Neurological disease, *n* (%)	6 (8)	6 (8)	0.001	1.00
Chronic renal disease, *n* (%)	7 (9)	11 (14)		0.45
Cirrhosis, *n* (%)	5 (6)	6 (8)		1.00
Lactate on admission, mEq/L	6.6 ± 5.1	8.8 ± 5.9		< 0.001
MV, *n* (%)	80 (100)	80 (100)		1.00
TTM, *n* (%)	25 (31)	70 (88)		< 0.001
Vasopressor therapy any time, *n* (%)	49 (62)	77 (96)		< 0.001
Inotropic therapy any time, *n* (%)	34 (43)	54 (68)		0.002
CRRT, *n* (%)	7 (9)	20 (25)		0.01
Coronary angiography, *n* (%)	21 (27)	35 (44)		0.03
PTCA/CABG, *n* (%)	12 (15)	19 (24)		0.22
Massive bleeding, *n* (%)	13 (16)	57 (71)		< 0.001
ICU stay, days	1 [1–3]	3 [1–10]		< 0.001
ICU survival, *n* (%)	14 (18)	18 (23)		0.46*
3-month favorable neurological outcome, *n* (%)	9 (11)	17 (21)		0.13*

*ICU* intensive care unit, *CA* cardiac arrest, *CPR* cardiopulmonary resuscitation, *ROSC* return of spontaneous circulation, *VF/VT* ventricular fibrillation/ventricular tachycardia, *COPD* chronic obstructive pulmonary disease, *ECPR* extracorporeal cardiopulmonary resuscitation, *CRRT* continuous renal replacement therapy, *MV* mechanical ventilation, *PTCA* percutaneous transluminal coronary angioplasty, *CABG* coronary artery bypass graft, *TTM* targeted temperature management, *PEA* pulseless electrical activity

*p value is reported for chi-square analysis

More patients in the ECPR group achieved ROSC than in the CCPR group (96% vs. 37%, *p* <  0.001). Patients treated with ECPR more frequently received TTM, vasopressor, or inotropic therapies; continuous renal replacement therapy (CRRT); and coronary angiography than CCPR patients. Massive bleeding was more frequent in the ECPR than in the CCPR group.

**Fig. 2:**
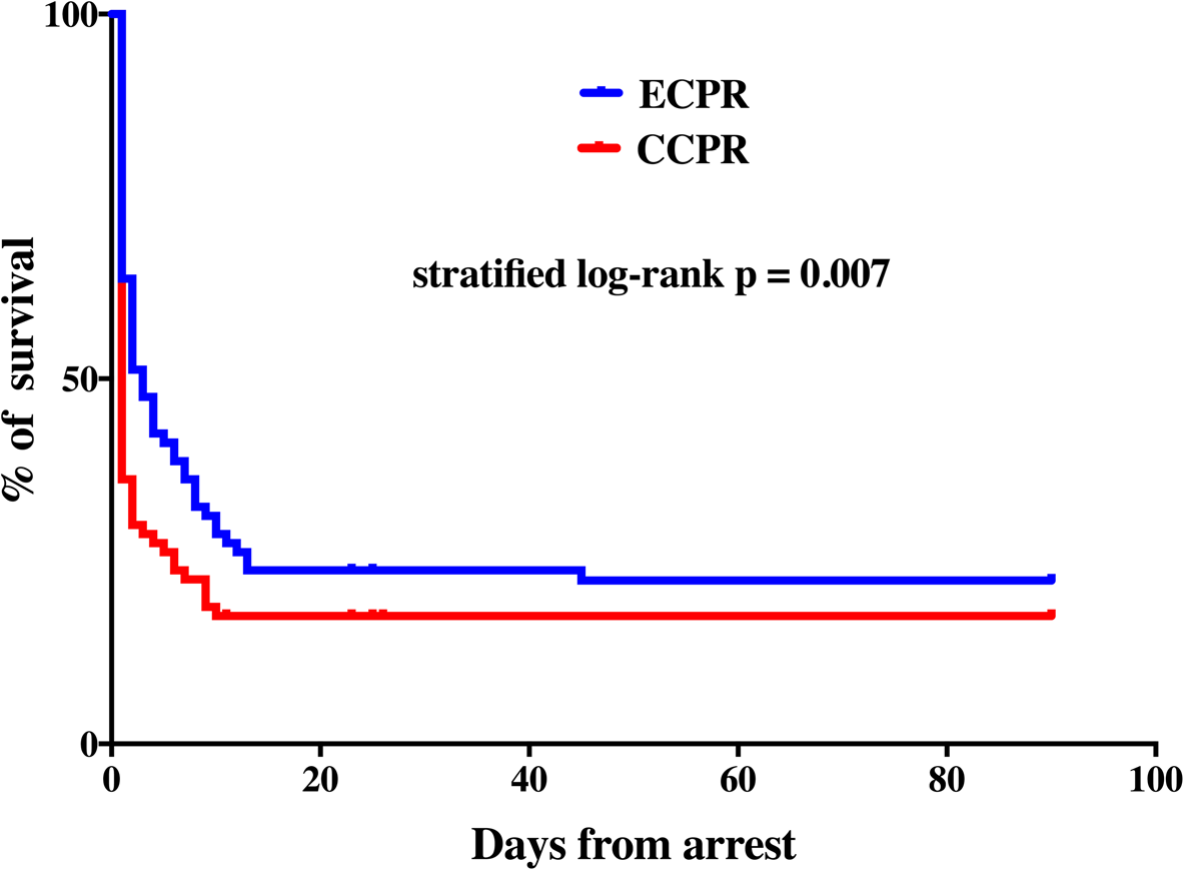
Kaplan-Meier plot of survival curves in the matched extracorporeal cardiopulmonary resuscitation (ECPR) and conventional cardiopulmonary resuscitation (CCPR) groups at 3 months. Differences in the time to survival between ECPR and CCPR were tested using stratified log-rank test and were quantified using Cox regression analysis stratified by matched pairs

**Fig. 3:**
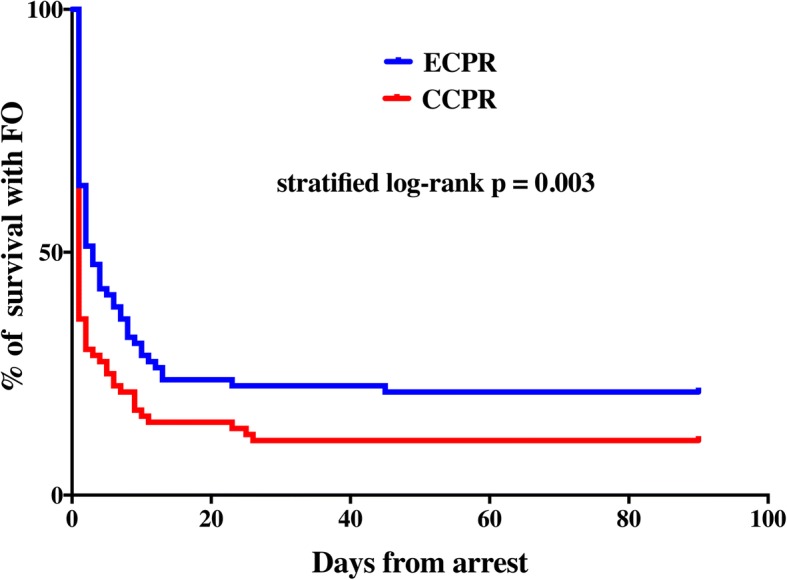
Kaplan-Meier plot of survival with favorable neurological outcome in the matched extracorporeal cardiopulmonary resuscitation (ECPR) and conventional cardiopulmonary resuscitation (CCPR) groups at 3 months. Differences in the time to survival with intact neurological outcome between ECPR and CCPR were tested using stratified log-rank test and were quantified using Cox regression analysis stratified by matched pairs

Survival rates were similar in the matched ECPR and CCPR groups (23% vs. 18%—conditional logistic regression: OR 1.40 [95% CIs 0.62–3.15], *p* = 0.42). Cox regression analysis stratified by matched pairs showed a significant increase in survival in the ECPR group (log-rank test *p* = 0.007; HR 1.71 [95% CIs 1.13–2.60]; Fig. 2). Seventeen patients (21%) in the matched ECPR group had a favorable neurological outcome compared to 9 (11%) in the matched CCPR group (conditional logistic regression: OR 1.75 [95% CIs 0.83–4.17], *p* = 0.11). Cox regression analysis stratified by matched pairs showed a significantly higher neurologic outcome rate in the ECPR group than in the CCPR group (log-rank test *p* = 0.003; HR 2.0 [1.5–5.3]; Fig. 3).

**Fig. 4:**
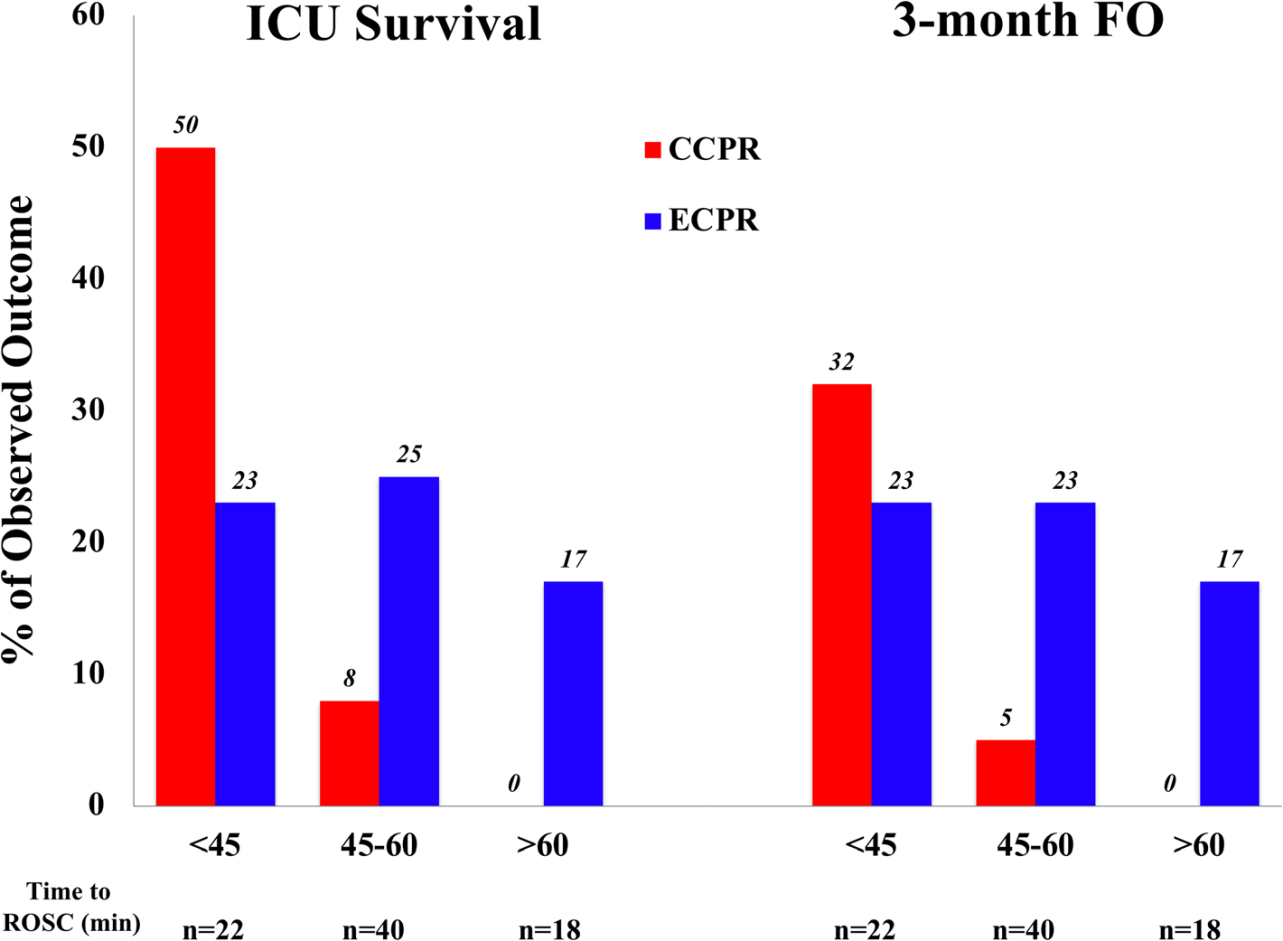
Relationship between time to return of spontaneous circulation (ROSC) and rate of ICU survival or favorable neurological outcome (FO) in the matched groups. ECPR extracorporeal CPR, CCPR conventional CPR

CCPR was associated with greater rates of survival and favorable neurological outcome in patients with shorter times to ROSC (< 45 min), whereas ECPR was associated with greater rates of survival and favorable neurological outcome in patients with longer times to ROSC (> 45 min) (Fig. 4). Subgroup analyses showed higher rates of ROSC in the ECPR group but similar outcomes between groups for all subgroups, except for a greater rate of ICU survival and of long-term favorable neurological outcome in ECPR patients with non-cardiac causes of arrest (Table 3). Among the patients with an initial non-shockable rhythm, 2 patients had a favorable neurological outcome in the CCPR group (both with a pulseless electrical activity, PEA) and 7 in the ECPR group (6 with PEA and 1 with asystole).

**Table 3:** Return of spontaneous circulation (ROSC), survival, and long-term neurological outcome among subgroups of patients. Data are presented as count (percentage)

	CCPR (*n* = 80)	ECPR (*n* = 80)	*p* value
OHCA	50	49	
ROSC, *n* (%)	26 (52)	47 (95)	< 0.001
ICU survival, *n* (%)	13 (26)	12 (24)	1.00
3-month FO, *n* (%)	8 (16)	12 (24)	0.34
IHCA	30	31	
ROSC, *n* (%)	4 (13)	30 (97)	< 0.001
ICU survival, *n* (%)	1 (3)	6 (19)	0.10
3-month FO, *n* (%)	1 (3)	5 (16)	0.19
Shockable rhythm	24	25	
ROSC, *n* (%)	16 (66)	23 (92)	0.04
ICU survival, *n* (%)	12 (50)	11 (44)	0.77
3-month FO, *n* (%)	7 (29)	10 (40)	0.55
Non-shockable rhythm	56	55	
ROSC, *n* (%)	14 (25)	52 (94)	< 0.001
ICU survival, *n* (%)	2 (4)	7 (13)	0.09
3-month FO, *n* (%)	2 (4)	7 (13)	0.09
Non-cardiac cause of arrest	44	22	
ROSC, *n* (%)	15 (34)	22 (100)	< 0.001
ICU survival, *n* (%)	2 (5)	5 (23)	0.04
3-month FO, *n* (%)	0 (0)	5 (23)	0.04
Cardiac cause of arrest	36	58	
ROSC, *n* (%)	15 (42)	55 (95)	< 0.001
ICU survival, *n* (%)	13 (36)	13 (22)	0.16
3-month favorable neurological outcome, *n* (%)	7 (19)	12 (20)	1.00

*OHCA* out-of-hospital cardiac arrest, *IHCA* in-hospital cardiac arrest, *ICU* intensive care unit

Of the 70 non-survivors at 3 months in the CCPR group, 57 died of “cardiac/multiple organ failure” and 13 of “neurological” reasons (*n* = 1 with brain death). One survivor had a CPC score of 3. Of the 63 non-survivors at 3 months in the ECPR group, 45 died of “cardiac/multiple organ failure” and 18 of “neurological” reasons (*n* = 5 brain death); within the “cardiac/multiple organ failure” causes, 3 patients had had full neurological recovery during the ICU stay but eventually died because of severe cardiogenic shock (*n* = 2) or severe hemorrhage after left ventricular assist device (LVAD) implantation (*n* = 1). In the CCPR group, 3 patients were candidates for organ donation (1 in brain death and 2 after circulatory death) and eventually underwent liver and kidney donation. In the ECPR group, 4 patients were candidates for organ donation (2 in brain death and 2 after circulatory death) and 3 of them eventually underwent liver and kidney donation.

## Discussion

In our experience of 635 patients with CA admitted to the ICU over a 5-year record period, our propensity score matched analysis showed a higher occurrence of long-term favorable neurological outcome in patients who received ECPR than those who received CCPR. Given the low survival rate with intact brain function, this suggests an almost 50% relative increase of good neurological recovery for ECPR than in CCPR.

Chen et al. also used a propensity score matching method in selected patients with IHCA who had had unsuccessful CPR for more than 10 min [11]; they reported a survival rate of 37% for ECPR, significantly higher than that for CCPR, although there was only a trend towards a better neurological outcome for ECPR-treated patients. In a second matched-control study conducted in IHCA patients, the survival rate in the ECPR group was also almost twice that of the CCPR group [12]. In one study focusing on OHCA, Maekawa et al. reported a higher survival rate in an ECPR group matched to a CCPR group (29% vs. 8%) [10]. Thus, our study, which included a greater cohort, is consistent with these previous observations. Whether this degree of favorable outcome is acceptable, considering the labor-intensive workload for the care team, or whether much higher improvement in survival and favorable neurological outcome rates are needed before proposing ECPR in this setting, is unclear. In a recent study, Yannopoulos et al. reported in 50 patients with refractory VF/VT undergoing ECPR that, despite similar times to ECMO as in our study, 42% of patients were discharged alive with intact neurological function, compared to 15% in their historical CCPR group [13]. However, only shockable rhythms were included, and these are associated with better outcomes than non-shockable rhythms, as also reported in our study. Moreover, the end-tidal CO_2_ measured at hospital admission was high (mean of 42 and 31 mmHg in survivors and non-survivors, respectively) [13], suggesting very high-quality CPR or recurrent VF/VT with intermittent cardiac recovery periods during resuscitation attempts. A recent systematic review showed that, although a trend towards improved survival with good neurologic outcome was reported in low risk of bias cohort studies, a high number of low-quality reports may overestimate the effect size of ECPR on survival among CA patients and high-quality randomized trials are urgently needed [14].

The use of a propensity score enabled us to minimize differences between groups and potential confounders that may have influenced the results, such as pre-admission comorbidities and the characteristics of CA. However, some differences persisted: ECPR patients had less frequently a non-cardiac cause of CA. Some studies have focused on the use of ECPR only for CA of cardiac origin. Although the aim of this study was not to specifically evaluate the impact of cause of CA on patient outcome, CAs of non-cardiac origin are in general associated with a worse outcome, unless the cause is potentially reversible, such as accidental hypothermia, myocarditis, or drug intoxication [15–17]. Nevertheless, as many patients in the CCPR group died before ROSC, it is possible that a cardiac cause (i.e., coronary ischemia and/or arrhythmias) of arrest might have not been recognized and then its occurrence underestimated. Importantly, we observed that 13% of patients in the ECPR group with a non-shockable initial rhythm also presented a favorable neurological outcome, mostly with a PEA. Although ECPR for patients with asystole has been often reported as futile, survival to discharge in patients with PEA as initial rhythm at the time of ECPR is around 15–20% [18] and patients with PEA should be considered for ECPR.

It remains very difficult to prospectively identify those patients who may benefit from ECPR. Clearly, one should aim to reduce the duration of CPR in order to minimize the risk of extensive anoxic brain damage, but whether earlier ECPR be of benefit to all patients it remains unknown. If ECMO cannulation is performed too early, patient who may otherwise have recovered on CCPR may be exposed unnecessarily to the potential complications of ECMO. In the present study, the mean time to ECPR was 54 min; Le Guen et al. showed that longer resuscitation period (i.e., up to 120 min) was associated with a survival rate of 5% in OHCA [19], so that initiation of ECMO should be considered between 30 and 75 min from arrest. Some groups have shown that ECPR performed in the ambulance may significantly shorten the time to ECPR and increase survival rates when compared to standard CPR [20]. Nevertheless, ECPR outside the hospital is even more complicated than in-hospital procedure. As such, a reduction of time to decision for hospital transport, improved quality of CPR—rather than the “scoop and run” provided in series from Japan—and shorter time to cannulate should be obtained to initiate ECPR in a safer environment, as the emergency department or the coronary angiography suite. In our study, one may argue that ECPR should be initiated only after 45 min, as it provides no significant benefits from shorter resuscitation time. However, the main message is that after 45 min of CCPR, the chances of survival and intact neurological outcome are extremely low (i.e., 2 out of 58 patients) and ECPR could be the only possibility to increase the chance of good outcome in this setting. Furthermore, the upper cutoff time for the duration of CCPR before ECPR that results in acceptable neurologic outcomes remains to be defined.

This study has some limitations. First, in terms of the impact on improved neurologic outcome after cardiac arrest, only selected patients were included. Over the 6-year period, only 112 patients were treated with ECPR. Second, data were retrospectively collected. Also, some “unmeasured variables,” such as the clinical status and appearance of the patient on admission or the decision why ECPR was not implemented (i.e., too late arrival, ECMO team not available, decision to transfer the patient to the hospital in case of OHCA), could not be included in the propensity score and might have influenced the final results. Similarly, it is impossible to consider whether a real choice or equipoise existed between ECPR and CCPR at the time point of treatment selection for each patient; only a randomized trial could potentially answer this question. Third, even though the propensity score reduced some selection bias, there may still have been some remaining bias because of the relatively small size of the matched groups and possible non-measured potential confounders (e.g., quality of ECPR or end-tidal CO_2_). Fourth, not all ECPR patients could be included in the final analysis, in particular because of prolonged CPR, which could not be matched in the CCPR group. Fifth, we observed no statistical difference in the absolute survival and favorable neurological outcome rates between groups, while the survival time with intact neurological function was significantly different between groups. This discrepancy was probably due to the relatively “small” number of patients in each group; moreover, it is important to notice that without ECMO most of patients with prolonged CCPR will never achieve ROSC and die immediately after admission, while one third of ECPR patients are still alive after 1 week and can potentially progress towards neurological and/or cardiac recovery or organ donation. Finally, the use of ECPR may result in bias because ECMO use cannot be blinded.

## Conclusions

ECPR may improve long-term neurological outcomes after CA compared to CCPR. Ongoing randomized trials in larger cohorts of patients may help us understand better which patients are most likely to benefit from this technique.

## Abbreviations

ALS: Advanced life support
CA: Cardiac arrest
CCPR: Conventional cardiopulmonary resuscitation
COPD: Chronic obstructive pulmonary disease
CPC: Cerebral Performance Category
CPR: Cardiopulmonary resuscitation
CRRT: Continuous renal replacement therapy
ECMO: Extracorporeal membrane oxygenation
ECPR: Extracorporeal cardiopulmonary resuscitation
EMS: Emergency medical system
IHCA: In-hospital cardiac arrest
LVAD: Left ventricle assist device
MAP: Mean arterial pressure
OHCA: Out-of-hospital cardiac arrest
ROSC: Return of spontaneous circulation
RRT: Renal replacement therapy
SMD: Standardized mean difference
TTM: Targeted temperature management
